# Supplementary material for: Late sporogonic stages of Plasmodium parasites are susceptible to the melanization response in Anopheles gambiae mosquitoes
Source: Front Cell Infect Microbiol. 2024 Aug 1;14:1438019. doi: 10.3389/fcimb.2024.1438019 (PMC11324593; doi:10.3389/fcimb.2024.1438019)
Supplement: Supplementary file 1 [file DataSheet_1.pdf]

Supplementary Material

Late sporogonic stages of *Plasmodium* parasites are susceptible to the melanization response in *Anopheles gambiae* mosquitoes

Suheir Zeineddine[1,3], Sana Jaber[1,3], Sally A. Saab[2], Johnny Nakhleh[2], George Dimopoulos[2], and Mike A. Osta[1*]

[1]Department of Biology, American University of Beirut, Beirut, Lebanon

[2]Harry Feinstone Department of Molecular Microbiology and Immunology, Bloomberg School of Public Health, Johns Hopkins University, Baltimore, MD 21205, USA

[*]**Correspondence:**

Mike A. Osta

mo07@aub.edu.lb

[3]These authors contributed equally to this work

Keywords: ***Anopheles gambiae***, ***Plasmodium***, **melanization, mosquito innate immunity,** ***Plasmodium*** **Sporogony**

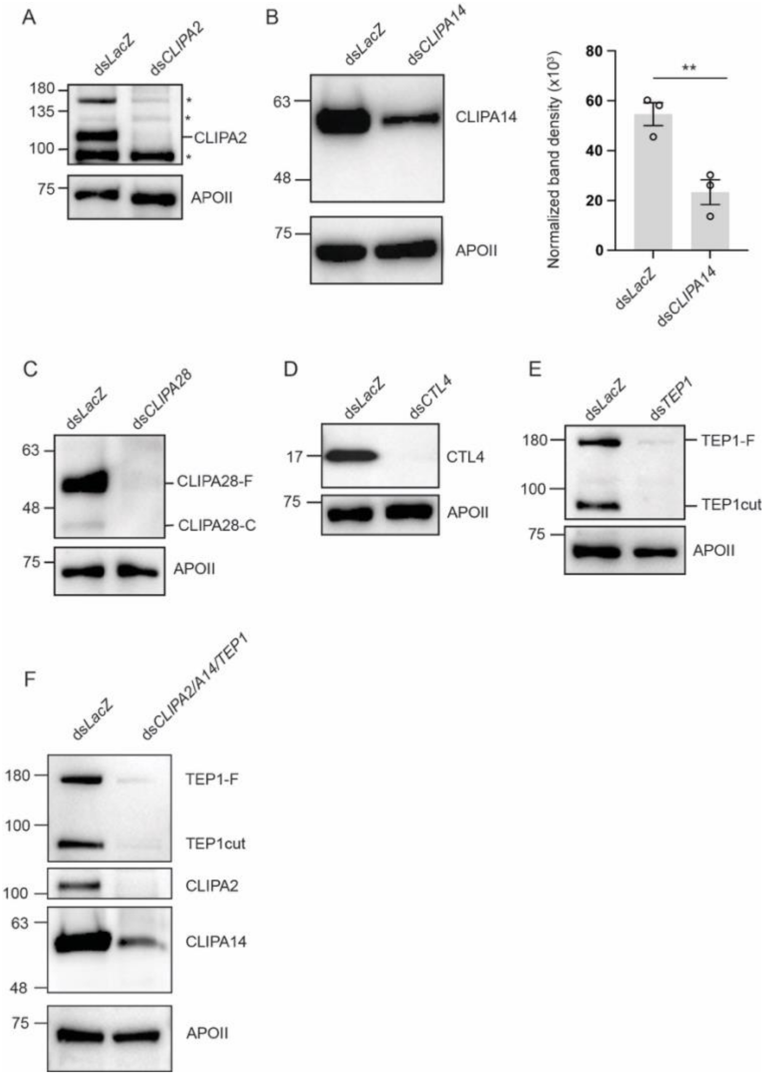

Supplementary Figure 1. Knockdown efficiency of RNAi-silenced genes. Shown are western blots of hemolymph samples extracted 7 days post-injection of mosquitoes with **(A)** ds*CLIPA2* (* asterisks indicate non-specific bands), **(B)** ds*CLIPA14* with a graph representing normalized CLIPA14 band density from 3 independent experiments, **(C)** ds*CLIPA28*, **(D)** ds*CTL4*, **(E)** ds*TEP1* and (**F**) ds*CLIPA2*/*A14*/*TEP1* (triple silencing). All hemolymph samples were extracted from 40 mosquitoes. Membranes were reprobed with αAPOII (without stripping) as loading control.

Supplementary Table 1

Raw data of Figure 1A: Independent experiments are shown in different colors

	dsLacZ		dsCLIPA2		dsCLIPA14		dsCLIPA2/A14	
gut #	live oocysts	melanized oocysts	live oocysts	melanized oocysts	live oocysts	melanized oocysts	live oocysts	melanized oocysts
1	3	0	7	1	3	0	43	22
2	33	0	8	0	3	1	14	1
3	29	0	7	0	1	0	16	17
4	237	0	0	0	0	0	95	141
5	14	0	1	0	114	0	9	0
6	10	0	54	0	2	0	1	0
7	39	0	34	1	267	1	0	1
8	81	0	5	0	4	3	89	13
9	5	1	2	0	32	0	113	8
10	1	0	5	0	247	2	0	8
11	12	0	7	0	2	0	12	8
12	6	0	4	0	0	0	6	2
13	78	0	0	0	1	2	96	57
14	30	0	246	0	21	1	66	54
15	52	0	5	0	52	2	10	0
16	210	0	15	0	22	2	10	0
17	327	0	6	0	65	4	149	45
18	514	0	167	0	8	0	43	101
19	325	0	1	0	32	0	150	10
20	40	0	14	0	13	2	78	57
21	436	0	53	0	126	16	0	0
22	0	0	0	0	12	6	81	31
23	14	0	149	0	41	0	288	16
24	5	0	215	1	1	0	67	59
25	15	0	34	0	23	7	46	1
26	0	0	0	0	25	9	118	40
27	15	0	36	0	0	0	9	20
28	64	0	0	0	442	0	12	21
29	262	0	0	0	87	30	0	0
30	77	0	4	0	26	0	0	0
31	13	0	0	0	2	0	17	0
32	0	1	0	0	12	0	0	4
33	0	1	137	0	34	0	21	30
34	31	0	1	0	14	0	0	0
35	0	0	8	0	62	5	8	5
36	5	0	0	0	2	0	0	0
37	27	0	0	0	2	0	0	1
38	10	0	7	0	4	0	2	0
39	3	0	0	0	30	10	0	0
40	4	0	0	0	0	0	0	2
41	48	0	0	0	0	1	15	24

42	0	1	7	0	0	0	0	0
43	3	0	0	0	2	3	13	1
44	8	0	0	0	17	0	1	2
45	21	0	0	0	77	0	0	0
46	0	0	0	0	71	2	34	5
47	7	0	2	0	2	0	88	11
48	7	0			58	0	24	2
49	1	5			10	7	2	0
50	26	0			0	0	0	0
51	16	0			0	0	15	7
52	89	0			0	0	46	15
53	0	0			15	0	5	15
54	0	0					70	75
55	28	0					0	1
56	0	0					1	0
57	0	0					59	8
58	1	0					43	0
59	9	0					23	27
60	0	0					7	0
61	4	0						
62	6	0						
63	0	4						

Raw data of Figure 1B: Independent experiments are shown in different colors

Gut #	dsLacZ live oocysts	dsLacZ melanized oocysts	dsCLIPA2/A14 live oocysts	dsCLIPA2/A14 melanized oocysts	dsCTL4 live oocysts	dsCTL4 melanized oocysts
1	25	0	16	0	1	0
2	20	2	14	0	3	0
3	28	0	4	1	134	0
4	114	3	19	33	19	0
5	5	0	16	2	15	0
6	93	0	19	50	3	0
7	4	0	5	0	30	1
8	39	0	54	0	86	0
9	7	0	9	3	1	0
10	2	0	1	1	2	0
11	10	0	17	1	23	0
12	4	0	8	0	37	0
13	158	0	6	3	0	0
14	15	3	0	0	7	0
15	61	0	13	12	1	0
16	49	0	4	1	1	0
17	42	0	1	0	5	0
18	34	0	3	10	0	0
19	1	0	6	28	0	0
20	28	1	0	0	5	0
21	144	0	153	8	0	0
22	4	0	8	0	34	0
23	0	0	88	16	10	0
24	4	0	1	1	37	2
25	14	2	1	2	3	0
26	3	0	0	0	12	0
27	24	0	14	11	4	0
28	145	0	69	0	150	0
29	36	0	85	1	56	0
30	4	0	30	34	0	0
31	0	0	6	2	9	0
32	2	0	53	11	54	1
33	67	0	12	13	11	5
34	4	0	43	62	6	0
35	1	0	14	20	4	0
36	72	0	32	0	87	0
37	12	0	25	65	33	0
38	14	0	23	3	8	0
39	13	0	5	14	17	0
40	21	0	6	14	240	0
41	15	0	60	14	0	2
42	2	0	0	2	31	0

43	54	0	30	39	3	0
44	41	0	12	7	18	0
45	7	0	4	0	106	0
46	14	0	1	3	6	0
47	25	0	14	63	0	0
48	4	0	41	10	23	0
49	250	0	6	2	1	0
50	1	0	2	0	16	0
51	7	0	34	50	1	0
52	36	0	0	0	2	0
53	21	0	47	65	0	0
54	131	1	9	8	26	0
55	30	0	16	0	103	0
56	18	0	6	1	0	0
57	15	0	2	0	39	0
58	0	0	3	5	25	0
59	40	0	13	1	11	0
60	26	0	0	0	23	0
61	54	0	18	31	55	0
62	9	0	3	2		
63	2	1	6	0		
64	55	0	20	5		
65	71	0	53	14		
66	73	0	19	9		
67	15	0	77	58		
68	74	0	19	8		
69	42	0	0	3		
70	40	0	14	0		
71	1	0	8	2		
72	111	0	1	3		
73	23	0	0	3		
74	225	0	1	0		
75	17	0	35	8		
76	3	0	107	0		
77			24	13		
78			21	50		
79			25	2		
80			7	0		
81			3	4		

Raw data of Figure 1E:Independent experiments are shown in different colors

	dsLacZ		dsCLIPA2/A14	
Gut #	live oocysts	melanized oocysts	live oocysts	melanized oocysts
1	5	0	0	0
2	25	0	34	0
3	73	0	15	1
4	4	0	18	0
5	6	0	50	0
6	107	0	0	0
7	35	0	0	0
8	6	0	5	0
9	21	0	200	0
10	6	0	0	0
11	0	0	0	0
12	0	0	22	0
13	0	0	6	0
14	1	0	116	0
15	53	0	32	0
16	142	0	142	0
17	4	0	230	0
18	0	0	7	0
19	40	0	11	0
20	65	0	26	1
21	56	0	15	0
22	18	0	103	0
23	44	0	254	0
24	4	0	244	0
25	88	0	100	3
26	160	0	36	0
27	70	0	93	0
28	82	0	34	1
29	1	0	89	0
30	0	0	110	12
31	6	0	10	0
32	147	0	49	0
33	86	0	0	0
34	130	0	9	0
35	141	0	65	0
36	173	0	66	1
37	31	0	0	0
38	43	0	0	0
39	84	0	0	0
40	85	0	32	0
41	132	0	3	0
42	260	0	11	0
43	33	0	162	0

44	1	0	158	0
45	19	0	28	0
46	0	13	0	2
47	152	0	2	0
48	22	0	3	0
49	0	0	17	3
50	12	0	0	0
51	0	0	0	0
52	30	0	15	0
53	0	0	0	0
54	0	0	124	0
55	142	0	1	0
56	1	0	0	0
57	151	0	0	0
58	31	0	73	0
59	0	0	22	0
60	83	0	96	0
61	186	0	0	0
62	29	0		
63	0	0		
64	0	0		
65	31	0		
66	0	0		
67	0	1		
68	40	0		
69	33	0		
70	3	0		
71	0	0		
72	0	0		
73	0	0		
74	46	0		
75	0	0		
76	0	0		
77	2	0		
78	1	0		
79	13	0		

Raw data of Figure 2A:Independent experiments are shown in different colors

Mosquito #	salivary gland sporozoites per mosquito		
	dsLacZ	dsCLIPA2/A14	dsCTL4
1	28500	0	500
2	0	0	0
3	500	0	1000
4	5500	0	1000
5	4500	0	0
6	500	0	22000
7	13500	0	0
8	500	500	500
9	0	500	5500
10	0	0	0
11	16500	12500	8000
12	0	7500	0
13	4000	6500	0
14	1000	8500	0
15	8000	5500	9000
16	3500	5500	7500
17	4500	7500	6000
18	9500	8000	9000
19	5000	2500	16500
20	6000	6500	7000
21	2000	7000	58500
22	24000	7000	13500
23	8500	3500	14000
24	6000	3500	20000
25	6000	6000	14500
26	3000	8500	13000
27	8500	500	18500
28	17500	500	15000
29	6500	0	16000
30	5500	1500	9500
31	6500	3500	2000
32	19000	1500	27500
33	15000	1500	22500
34	31000	0	9000
35	84500	500	6500
36	16000	500	17000
37	26500	500	6500
38	9000	12000	45000
39	80000	7000	15500
40	16500	11500	14500
41	33500	0	87500
42	22500	0	32000

43	56000	0	23500
44	29500	0	27500
45	37000	0	122000
46	100000	0	14500
47	11000	0	16500
48	32500	0	74500
49	27000	0	13000
50	23000	0	27000
51	53000	0	14500
52	9500		13500
53	20500		0
54	28500		0
55	0		16000
56	0		1500
57	0		5000
58	0		0
59	9500		0
60	1000		10500
61	1500		0
62	39000		0
63	500		0
64	500		0
65	19500		0
66	0		0
67	0		0
68	35500		54500
69	2000		0
70	0		0
71	0		3000
72	0		0
73			3000
74			1500
75			0
76			33500
77			1000
78			500
79			0

Raw data of Figure 3: Independent experiments are shown in different colors

	Sugar fed				Extra blood meal			
	**dsLacZ**		**dsCLIPA2/A14**		**dsLacZ**		**dsCLIPA2/A14**	
Gut #	live oocysts	melanized oocysts	live oocysts	melanized oocysts	live oocysts	melanized oocysts	live oocysts	melanized oocysts
1	10	0	53	1	0	0	24	47
2	42	2	1	0	147	0	23	7
3	11	0	33	5	0	0	1	0
4	81	0	0	0	0	0	2	2
5	40	0	2	1	1	0	0	0
6	0	0	5	7	12	11	14	18
7	2	0	42	0	84	1	15	4
8	13	0	5	8	7	0	43	112
9	200	0	12	31	37	3	8	9
10	35	0	16	0	11	0	14	24
11	2	0	2	0	8	0	5	0
12	80	0	101	5	6	0	203	23
13	180	0	22	0	0	0	0	0
14	210	0	15	15	2	0	0	0
15	0	0	134	18	37	2	43	4
16	11	0	0	0	15	0	2	0
17	1	0	1	1	90	1	23	7
18	94	0	1	5	0	0	2	0
19	80	1	4	0	20	0	2	0
20	73	0	14	27	8	0	151	19
21	67	0	71	15	26	0	85	74
22	27	0	62	22	0	0	3	0
23	14	0	30	6	8	0	2	0
24	0	0	22	29	8	0	1	13
25	3	0	250	30	11	0	17	10
26	73	0	49	22	2	0	52	30
27	44	0	15	11	1	1	26	28
28	1	0	14	19	12	0	21	28
29	16	0	17	6	8	1	0	0
30	19	0	2	0	0	0	64	139
31	3	0	0	1	0	0		
32	6	0			67	0	30	96
33	12	0	3	1	0	0	15	20
34	20	0	62	39	11	0	6	4
35	4	0	9	18			31	32
36	39	0	46	43			41	20
37	10	0	116	50			54	32
38	23	0	5	6			2	2
39	5	0	18	37			61	62
40	0	0	40	10			0	0
41	67	0	20	19			2	2

42	80	0	10	0	72	42
43	160	0	126	28	0	0
44	23	0	7	61	32	52
45	15	0	1	1	22	2
46	19	0	5	18	9	54
47	35	0	85	69	1	10
48	14	0	21	12	13	21
49			0	0	0	0
50			14	1	2	6
51					18	0
52					77	74
53					51	75
54					117	65
55					38	96
56					3	27
57					6	12
58					16	34
59					0	0
60					8	5
61					74	128
62					4	20
63					1	5
64					22	29
65					16	47
66					18	15
67					43	44

Raw data of figure 4A:Independent experiments are shown in different colors

	**dsLacZ**		**dsCLIPA2/A14**		**dsCLIPA2/A14/A28**		**dsCLIPA2/A14/TEP1**	
Gut #	live oocysts	melanized oocysts	live oocysts	melanized oocysts	live oocysts	melanized oocysts	live oocysts	melanized oocysts
1	68	0	9	15	26	0	104	0
2	58	1	13	7	284	0	2	0
3	140	0	10	0	111	0	1	0
4	339	0	3	1	100	0	17	0
5	16	0	113	5	125	0	67	0
6	14	0	39	77	144	3	1	0
7	82	0	17	34	38	0	4	0
8	85	2	1	0	29	0	0	2
9	89	0	4	0	0	0	102	7
10	3	0	92	20	0	0	8	0
11	133	0	50	36	52	0	77	0
12	1	0	35	22	54	0	48	0
13	26	0	0	0	163	0	4	14
14	13	0	8	0	66	0	0	0
15	65	0	10	20	140	0	36	0
16	121	0	7	8	24	0	19	0
17	27	0	57	30	106	0	108	1
18	0	0	26	1	4	0	0	0
19	39	0	0	0	42	1	7	0
20	2	0	13	0	6	0	4	1
21	1	0	13	14	4	2	35	1
22	32	0	38	80	34	0	7	1
23	3	3	8	21	1	2	13	0
24	102	0	6	25	5	0	11	0
25	36	0	26	6	1	0	72	0
26	1	0	7	0	1	1	9	1
27	0	0	11	11	16	0	29	0
28	14	0	9	1	8	0	1	0
29	3	0	2	16	2	0	4	0
30	24	1	3	3	5	0	108	0
31	35	0	18	29	34	0	2	0
32	64	0	0	0	82	0	2	0
33	6	0	2	21	35	1	22	1
34	15	0	0	0	15	0	2	0
35	16	0	33	50	89	0	3	0
36	4	0	32	3	97	1	25	1
37	24	0	1	5	87	0	5	2
38	3	0	32	30	115	0	0	0
39	0	0	29	33	11	0	8	0
40	6	0	2	8	1	0	46	0
41					39	0	12	0
42					19	0	10	0

43	10	0	3	0
44	0	0	12	0
45	4	0	5	1
46	48	0	5	0
47	31	0	42	0

Raw data of figure 5A: *P. falciparum* early stage melanization (low median infection)

Gut #	dsGFP L	dsGFP M	dsA2/A14 L	dsA2/A14 M
1	24	0	0	0
2	161	0	109	2
3	167	0	15	0
4	167	0	123	0
5	100	0	200	0
6	190	0	0	0
7	1	0	20	0
8	5	0	3	0
9	2	0	0	0
10	2	0	0	0
11	1	0	50	0
12	0	0	79	0
13	17	0	36	0
14	239	0	2	0
15	160	0	188	0
16	0	0	22	0
17	0	0	26	0
18	91	0	175	0
19	78	0	35	0
20	130	0	7	0
21	0	0	7	0
22	56	0	95	0
23	0	0	0	0
24	70	0	105	0
25	0	0	230	0
26	123	0	195	0
27	140	0	40	0
28	120	0	24	0
29	128	0	0	0
30	0	0	160	4
31	200	0	30	0
32	0	0	84	1
33	26	0	10	0
34	73	0	0	0
35	16	0	0	0
36	0	0	4	0
37	35	0	0	0
38	46	0	45	0
39	1	0	70	5
40	0	0	0	0
41	1	0	3	0
42	14	0	0	0
43	1	0	24	0

44	179	0	3	0
45	35	0	5	0
46	0	0	0	1
47	17	0	18	0
48	0	0	40	0
49	85	0	11	0
50	58	0	15	0
51	0	0	12	0
52	0	0	4	0
53	78	0	24	0
54	7	0	8	0
55	25	0	0	0
56	14	0	16	0
57	1	0	1	0
58	4	0	9	0
59	90	0	34	0
60	7	0	12	0
61	12	0	0	0
62	13	0	0	0
63	2	0	30	0
64	0	0	26	0
65	1	0	0	0
66	20	0	24	0
67	10	0	0	0
68	0	0	21	0
69	4	0	23	0
70	19	0	11	0
71	10	0	5	0
72	8	0	11	0
73	3	0	41	0
74	3	0	0	0
75	7	0	3	0
76	18	0	6	0
77	1	0	12	0
78	4	0	1	0
79	26	0	6	0
80	4	0	23	0
81	0	0	18	0
82	0	0	1	0
83	46	0	5	0
84	12	0	5	0
85	2	0	19	0
86	33	0	0	0
87	18	0	13	0
88	0	0	9	0
89	8	0	0	0

90	7	0	0	0
91	40	0	5	0
92	4	0	19	0
93	4	0	3	0
94	5	0	6	0
95	1	0	5	0
96	0	0	6	0
97	5	0	2	0
98	7	0	16	0
99	0	0	6	0
100	5	0	9	0
101	5	0	0	0
102	18	0	10	0
103	14	0	11	0
104	18	0	42	0
105	9	0	6	0
106	15	0	9	0
107	4	0	26	0
108	6	0	0	0
109	8	0	1	0
110	1	0	10	0
111	3	0	1	0
112	22	0	0	0
113	1	0	7	0
114	0	0	9	0
115	0	0	2	0
116	4	0	5	3
117	1	0	37	0
118	1	0		
119	0	0		
120	0	0		
121	5	0		
122	16	0		
123	4	0		
124	6	0		
125	4	0		
126	0	0		
127	21	0		

Raw data of figure 5B: *P. falciparum* early stage melanization (high median infection)

Gut #	dsGFP L	dsGFP M	dsA2/A14 L	dsA2/A14 M
1	2	0	156	1
2	11	0	65	0
3	47	0	126	1
4	60	0	158	0
5	217	0	0	0
6	0	0	82	0
7	173	0	224	0
8	17	0	101	2
9	57	0	4	0
10	94	0	114	0
11	8	0	17	0
12	143	0	30	0
13	142	0	60	0
14	57	0	25	0
15	14	0	40	0
16	147	0	126	1
17	0	0	17	0
18	12	0	173	0
19	99	0	116	1
20	109	0	83	0
21	104	0	68	0
22	102	0	68	0
23	64	0	134	0
24	50	0	161	3
25	142	0	181	2
26	0	0	3	0
27	114	0	65	0
28	180	0	97	0
29	7	0	212	0
30	129	0	69	0
31	145	0	87	0
32	27	0	35	0
33	50	0	0	0
34	92	0	41	0
35	75	0	63	0
36	76	0	23	0
37	43	0	36	0
38	60	0	40	0
39	170	0	102	0
40	160	0	98	0
41	50	0	7	0
42	64	0	43	0
43	30	0	165	0

44	76	0	94	0
45	53	0	74	0
46	8	0	40	0
47	70	0	66	0
48	50	0	63	1
49	55	0	5	0
50	42	0	3	0
51	70	0	105	1
52	62	0	68	0
53	68	0	70	0
54	68	0	58	0
55	148	0	7	0
56	34	0	84	0
57	132	0	57	0
58	0	0	75	0
59	102	0	100	1
60	40	0	54	0
61	38	0	26	0
62	78	0		
63	140	0		

Raw data of figure 5C: *P. falciparum* late stage melanization (low median infection)

Gut #	dsGFP L	dsGFP M	dsA2/A14 L	dsA2/A14 M
1	18	0	9	1
2	20	0	12	1
3	15	0	6	0
4	1	0	0	1
5	2	0	0	0
6	18	0	16	0
7	2	0	2	1
8	30	0	13	2
9	37	0	0	0
10	25	0	17	2
11	38	0	0	0
12	0	0	12	1
13	0	0	4	0
14	0	0	6	0
15	0	0	4	0
16	0	0	90	0
17	4	0	0	0
18	38	0	0	0
19	32	0	13	0
20	4	0	41	0
21	11	0	0	0
22	21	0	1	0
23	0	0	4	0
24	44	0	16	0
25	38	0	45	1
26	35	0	44	0
27	25	0	2	0
28	26	0	29	0
29	14	0	31	0
30	30	0	63	0
31	1	0	57	0
32	30	0	8	7
33	62	0	41	3
34	25	0	52	0
35	31	0	31	1
36	15	0	36	2
37	11	0	34	0
38	97	0	24	3
39	35	0	0	0
40	17	0	1	1
41	21	0	11	1
42	22	0	50	2
43	37	0	53	1

44	31	0	40	0
45	17	0	72	0
46	7	0	57	0
47	8	0	20	3
48	27	0	55	2
49	54	0	46	4
50	5	0	3	0
51	51	0	35	4
52	20	0	57	2
53	93	0	7	0
54	39	0	31	3
55	9	0	84	5
56	74	0	19	13
57	3	0	50	12
58	59	0	112	9
59	6	0	34	21
60	57	0	58	2
61	24	0	33	2
62	23	0	109	12
63	29	0	1	0
64	34	0	0	0
65	20	0	63	2
66	22	0	24	0
67	7	0	42	7
68	42	0	74	2
69	2	0	0	0
70	60	0	56	3
71	71	0	4	0
72	20	0	19	3
73	22	0	4	4
74	26	0	4	3
75	6	0	0	0
76	5	0	11	0
77	0	0	0	0
78	21	0	8	0
79	6	0	2	1
80	7	0	8	0
81	6	0	0	0
82	21	0	1	0
83	2	0	0	0
84	16	0	1	6
85	43	0	0	0
86	31	0	0	0
87	15	0	1	0
88	31	0	2	0
89	3	0	0	0

90	0	0	0	0
91	0	0	1	1
92	12	0	0	2
93	38	0	7	0
94	3	0	0	2
95	8	0	3	0
96	0	0	3	0
97	1	0	29	3
98	11	0	0	0
99	2	0	0	7
100	3	0	0	3
101	9	0	11	4
102	5	0	1	0
103	0	0	0	2
104	5	0	0	3
105	19	0	1	4
106	0	0	0	0
107	4	0	0	0
108	1	0	0	0
109	9	0	3	1
110	7	0	0	0
111	8	0	0	0
112	6	0	0	0
113	19	0	0	0
114	4	0	5	0
115	2	0	14	0
116	0	0	2	0
117	6	0	2	0
118	2	0	3	0
119	0	0	0	1
120	0	0	7	1
121	4	0	4	0
122	7	0	2	0
123	3	0	1	0
124	19	0	4	0
125	1	0	10	0
126	1	0	3	0
127	1	0	0	0
128	0	0	0	0
129	2	0	7	0
130	0	0	2	0
131	13	0	2	0
132	7	0	0	0
133	0	0	1	0
134	8	0	0	0
135	7	0	5	1

136	0	0	3	1
137	3	0	0	0
138	4	0	0	0
139	14	0	0	0
140	0	0	3	0
141	2	0	6	1
142	2	0	0	0
143	2	0	2	0
144	0	0		
145	2	0		
146	1	0		
147	7	0		
148	4	0		
149	1	0		
150	1	0		
151	1	0		
152	5	0		
153	3	0		
154	1	0		
155	8	0		
156	6	0		
157	15	0		
158	0	0		
159	2	0		
160	14	0		
161	0	0		
162	5	0		
163	0	0		
164	0	0		
165	1	0		
166	0	0		
167	5	0		

Supplementary Table 2. Primers used for dsRNA production

Gene	Primer sequence (T7 promoter sequence underlined)	Reference
LacZ	For: 5'-TAATACGACTCACTATAGGGAGAATCCGACGGGTTGTTACT-3'<br>Rev: 5'-TAATACGACTCACTATAGGGCACCACGCTCATCGATAATTT-3'	(Habtewold et al., 2008)
GFP	For: 5’- TAATACGACTCACTATAGGGTTCATCTGCACCACCGGC-3’<br>Rev: 5’-TAATACGACTCACTATAGGGCTGGTAGTGGTCGGCGAG-3’	(Simoes et al., 2017)
CLIPA2<br>(AGAP011790)	For:5'-TAATACGACTCACTATAGGGATCCTAACAACGGCACACTGTGTGA-3'<br>Rev:5'-TAATACGACTCACTATAGGGTCCTGATCGCCATGATTGGTGGTGCT-3'	(Yassine et al., 2014)
CLIPA14<br>(AGAP011788)	For: 5'- TAATACGACTCACTATAGGGCGGCATCATCGACATCCGTGTC-3'<br>Rev: 5'- TAATACGACTCACTATAGGGGTTGCTGTCGGCGACACGCTCCT-3'	(Nakhleh et al., 2017)
CLIPA28<br>(AGAP010730)	For: 5'-TAATACGACTCACTATAGGGAGACCACCAAGGAACCGTTCCCGCAGCAA-3'<br>Rev: 5'-TAATACGACTCACTATAGGGAGACCGCAACCGATGCCCCACGATACGAT-3'	(El Moussawi et al., 2019)
TEP1<br>(AGAP010815)	For: 5'-TAATACGACTCACTATAGGGTTTGTGGGCCTTAAAGCGCTG-3'<br>Rev: 5'- TAATACGACTCACTATAGGGACCACGTAACCGCTCGGTAAG-3'	(Povelones et al., 2011)
CTL4<br>(AGAP005335)	For: 5'- TAATACGACTCACTATAGGGGTTAGCAGCATTGGGATTACCCT-3'<br>Rev: 5'- TAATACGACTCACTATAGGGGAAGTCGCAACCCAGCTCATTGT-3'	(Povelones et al., 2013)

**Supplementary Table 3. Infection data for *P. falciparum* experiments**					
Experiment	Silenced gene	parasite	N	Median L, M	Prevalence %
Early-stage melanization (Low median)	GFP	Pf NF54	127	6, 0	79
	A2/A14	Pf NF54	117	9, 0	79
Early-stage melanization (High median)	GFP	Pf NF54	63	64,0	94
	A2/A14	Pf NF54	61	68,0	97
Late stage melanization	GFP	Pf NF54	167	7,0	84
	A2/A14	Pf NF54	143	4,0	77

References

El Moussawi, L., Nakhleh, J., Kamareddine, L. and Osta, M. A. (2019). The mosquito melanization response requires hierarchical activation of non-catalytic clip domain serine protease homologs. *PLoS Pathog.* 15**,** e1008194. doi: 10.1371/journal.ppat.1008194

Habtewold, T., Povelones, M., Blagborough, A. M. and Christophides, G. K. (2008). Transmission blocking immunity in the malaria non-vector mosquito *Anopheles quadriannulatus* species A. *PLoS Pathog.* 4**,** e1000070.

Nakhleh, J., Christophides, G. K. and Osta, M. A. (2017). The serine protease homolog CLIPA14 modulates the intensity of the immune response in the mosquito Anopheles gambiae. *J. Biol. Chem.* 292**,** 18217-18226. doi: 10.1074/jbc.M117.797787

Povelones, M., Bhagavatula, L., Yassine, H., Tan, L. A., Upton, L. M., Osta, M. A. and Christophides, G. K. (2013). The CLIP-Domain Serine Protease Homolog SPCLIP1 Regulates Complement Recruitment to Microbial Surfaces in the Malaria Mosquito Anopheles gambiae. *PLoS Pathog.* 9**,** e1003623. doi: 10.1371/journal.ppat.1003623

Povelones, M., Upton, L. M., Sala, K. A. and Christophides, G. K. (2011). Structure-function analysis of the Anopheles gambiae LRIM1/APL1C complex and its interaction with complement C3-like protein TEP1. *PLoS Pathog.* 7**,** e1002023.

Simoes, M. L., Mlambo, G., Tripathi, A., Dong, Y. and Dimopoulos, G. (2017). Immune Regulation of Plasmodium Is Anopheles Species Specific and Infection Intensity Dependent. *MBio.* 8. doi: 10.1128/mBio.01631-17

Yassine, H., Kamareddine, L., Chamat, S., Christophides, G. K. and Osta, M. A. (2014). A serine protease homolog negatively regulates TEP1 consumption in systemic infections of the malaria vector Anopheles gambiae. *J. Innate Immun.* 6**,** 806-18. doi: 10.1159/000363296
